# Computational phosphoproteomics: From identification to localization

**DOI:** 10.1002/pmic.201400372

**Published:** 2015-02-17

**Authors:** Dave C H Lee, Andrew R Jones, Simon J Hubbard

**Affiliations:** 1 Faculty of Life Sciences, University of ManchesterManchester, UK; 2 Institute of Integrative Biology, University of LiverpoolLiverpool, UK

**Keywords:** Bioinformatics, Data processing and analysis, Phosphoproteomics, Technology

## Abstract

Analysis of the phosphoproteome by MS has become a key technology for the characterization of dynamic regulatory processes in the cell, since kinase and phosphatase action underlie many major biological functions. However, the addition of a phosphate group to a suitable side chain often confounds informatic analysis by generating product ion spectra that are more difficult to interpret (and consequently identify) relative to unmodified peptides. Collectively, these challenges have motivated bioinformaticians to create novel software tools and pipelines to assist in the identification of phosphopeptides in proteomic mixtures, and help pinpoint or “localize” the most likely site of modification in cases where there is ambiguity. Here we review the challenges to be met and the informatics solutions available to address them for phosphoproteomic analysis, as well as highlighting the difficulties associated with using them and the implications for data standards.

## 1 Introduction

Phosphorylation is a PTM that is deeply embedded in the cellular system architecture. Its role, either directly or indirectly, is regulatory where it acts to relay external stimuli to specific and carefully evolved cascades of events that evoke appropriate biological responses. For example, a single kinase, such as CDK1, can trigger hundreds of time-resolved downstream events, all ultimately controlled by this master regulator through independent phosphorylations [1]. The prevalence of phosphorylation in signaling and regulatory processes has been widely cited to affect 30% of the proteome [2,3]. However, with the growing volume and quality of data being generated by the phosphoproteomics community, this long-standing estimate might well represent an underestimate given the increasingly comprehensive annotation of the total phosphoproteome [4].

The integral role of phosphorylation in mediating multiple crucial biological events [4–9] has led to major effort into methods and technologies capable of fully elucidating the “phosphoproteome,” the site-level resolution of phosphorylation of the proteome under a given condition. Given the advances in analytical capability in recent years and the increasing interest in mapping the mechanistic detail of intracellular signaling pathways, phosphoproteomics has become an active field with many groups attempting to find candidate targets for kinases and phosphatases of interest. Although there are many individual studies based around antibodies to monitor and validate phosphorylation status of individual sites [10], for high-throughput and genome-wide studies the analytical method of choice is typically MS (cf. [8,11]). This has been driven by the ever-improving instrumentation and associated analytical chemistry, in particular MS augmented by MS-compatible phosphorylation enrichment techniques [12–14], which have made this strategy a key player in the field, enabling high volumes of data to be produced with high (and constantly improving) resolution and precision, exemplified recently by some landmark studies [8,11,15,16]. The increased prevalence of such studies has resulted in a deluge of data that in turn has supported the rapid expansion of content contained within phosphorylation databases [17–20]. Notably, these databases have been an invaluable resource in the development of phosphorylation site predictors, which predict the phosphorylation status of candidate sites using information derived from the immediate surrounding residues [21–23] or three-dimensional environment [24] where they provide the necessary data required for building, training, and optimization [25].

Although MS-based approaches are popular, like most high-throughput technologies they also have their limitations. In particular for phosphoproteomics, there are several key challenges to be overcome in a MS-based experiment. First, phosphopeptides captured by phosphorylation enrichment need to be ionized and analyzed in the mass spectrometer to generate MS/MS. The identities of the underlying sequence for each of these MS/MS should then be deduced using computational tools. Individual spectra generate multiple candidate peptide spectrum matches (PSMs), usually ranked by a search engine score. Here, the principal issue is to associate a unique peptide match to each spectrum with an associated level of statistical significance (i.e., *p*-value or false discovery rate (FDR)) in order to minimize false positives—this is the so-called “identification” challenge. Redundancy is generated from multiple PSMs to the same peptide sequence, and care should be taken to estimate significance at the peptide as well as PSM level. Equally, protein inference is also challenging when peptide sequences map to multiple parent proteins, leading to challenges when integrating scores and statistical significance to the protein level.

Second, if a phosphopeptide has been confidently identified, then there may be ambiguity in the true site (or sites) of phosphorylation as a given peptide may have multiple residues that could be modified, and indeed in some cases, it may be possible for multiple independent sites to be modified. Hence, it is often necessary to decide between different phosphoisomers—this is the so-called “localisation” challenge.

Third, it is usually desirable to quantify the stoichiometry of phosphorylation compared to other isoforms, since subtle changes in phosphorylation level are believed to lead to large changes in downstream signaling. This “quantification” challenge is substantial, since ideally one would be able to quantify not only the level of the phosphopeptide, but also changes in this in the context of changes in the overall protein level and all its phosphoisoforms. This is evident when considering such systems as cell-cycle control kinases where different phosphoisoforms have different affinities for other cyclin-kinase pairs and subtle shifts in these properties are tightly couple to regulation of the cell cycle itself [1,26].

Finally, this leads on to whether the phosphorylation sites identified are truly functional. Presently, the most popular strategy employed to assign functional significance is via SILAC, where the functional status of phosphosites is typically assumed based on them meeting a minimal arbitrary fold-change in a quantitative experiment between kinase/phosphatase active and inactive conditions [27–31].

In this review, we discuss the various issues involved in applying informatic pipelines to identify and for analyzing experimental phosphoproteomic data. Our focus is to make the reader aware of why identification and localization are such daunting tasks and the remaining outstanding questions that the field is presently working toward solving.

## 2 Benchmarking studies highlight inconsistencies in phosphopeptide informatics

The challenges presented by phosphoproteomics to informatics tools were the focus of a 2010 study conducted by the Proteome Informatics Research Group (iPRG) of the Association of Biomolecular Resource Facilities (ABRF) (http://www.abrf.org/index.cfm/group.show/ProteomicsInformaticsResearchGroup.53.htm#943). In this study, several groups were provided with the same set of MS/MS from an enriched phosphorylation sample derived from different chromatography fractions and asked to analyze and return the set of statistically significant identifications, and if possible, confidently localized sites. No restrictions on informatic tools and strategies were placed on groups regarding how they analyze the data and it is this point that was the main objective of the study, to assess the degree of conformity between groups with regards to how the data are handled and, more importantly, the identification and localization outcomes. Although the precise identities of the phosphopeptides and sites of phosphorylation were not known a priori, the results were still highly variable. Indeed, on average, a 57% agreement was found between the sets of phosphorylated peptides identified between groups when considered on a pairwise basis. However, perhaps more worryingly, this level of agreement decreased substantially to ∼38% consensus agreement when considering site localization. These results are important, since although it was not possible to judge the absolute accuracy of the results there was clear disagreement between groups, demonstrating that informatic workflows on the same data following stringent statistical thresholds resulted in dramatically different outcomes. Furthermore this represents a “real-world” example where the only sources of variance are the experience and knowledge of the researchers, and the choice and method of applying informatics pipelines. The study also highlighted that different groups using the same basic pipelines can achieve different, conflicting results. Given that these were largely expert groups, it suggests that false-positive and false-negative rate in high-throughput phosphoproteomic data sets could be substantial and that best practice is still to be defined.

## 3 Identification of phosphopeptides: Challenges and issues

A typical shotgun or high-throughput proteomics experiment targets the precursor ions eluting into the mass spectrometer for fragmentation in order to generate product ions that are characteristic of the precursor and which form the basis of peptide identification by sequence database search or de novo sequencing. The peptides are first generated from a digestion of the denatured proteins under study, typically with the endopeptidase trypsin, prior to mass spectrometric analysis. The subsequent identification step of the MS/MS spectra generated relies on informatics tools, predominantly database search engines (cf. [32–34]), as well as to a lesser extent de novo sequencers (cf. [35–37]) and search tools based on spectral libraries [38]. For the majority of data-dependent acquisition strategies in shotgun proteomics where the identity of the peptides is not known a priori, database search engines are the most widely used in phosphoproteomic analyses. A large number of such tools exist, well-reviewed recently by Eng and colleagues [39].

Unfortunately, because candidate PSMs from any identification tool will contain false matches, a measure of confidence needs to be assigned to select true identifications while avoiding those that are incorrect. Proteomics as a field has generated a variety of means to assess the levels of potential false discovery, specifically in sets of PSMs. Presently, the most widely used approaches compute a FDR or FDR-related statistic such as *q*-values [40,41], allowing the user to control for the expected number of false-positive identifications. For most database search engines, empirical FDRs are computed through the target-decoy strategy where MS/MS are searched against the sequence database and a “decoy” version of it [42,43]. The formation of the “decoy” set is most commonly done *via* direct reversal for simplicity, but other methods exist (e.g., randomly shuffled, database generated from residue frequencies), and is either concatenated with the target database or run separately. The precise structure of how to build the appropriate FDR model has been subjected to vigorous assessment to find an optimal strategy [43–45].

Another possible issue stems from the use of mixed sets of both modified and unmodified peptide spectra when determining the FDR. This is relevant for phosphoproteomics and has recently been discussed in the literature [46,47]. Here, it is postulated that FDR calculations should formally consider the phosphopopulation independently, reasoning that the underlying physicochemical properties of the phosphorylated peptides are different from the nonphosphorylated ones leading to different characteristic fragmentation behaviors (such as dominant neutral loss of the phosphate described previously) and by extension phosphospecific and nonphospho-specific scoring distributions. However, although this could be a potential issue, most phosphoproteomic studies are conducted on highly enriched peptide sets where this is unlikely to be a dominating factor.

Here we focus on issues that are specific to phosphopeptide identification, which are additional challenges to the identification and assessment of significance in mixed populations of modified and unmodified peptides. A common issue is that phosphorylation can hinder comprehensive identification of a phosphopeptide by lowering the ionization efficiency [3]. Here, the presence of the phosphate contributes a negative charge to an otherwise positively charged peptide (under acidic conditions), which interferes with the ionization process into the gas phase. This reduction in the phosphopeptide population makes for a potent obstacle by proportionally reducing the corresponding product ions required for successful identification. Multiple phosphates can exacerbate this issue [48] while multiple protons may rectify it [3].

The labile nature of the phosphate group itself is also an issue, as it has a lower activation energy threshold than an amide bond and is frequently lost as a neutral species from the precursor ion during fragmentation in the gas phase. This reduces the level of fragmentation in the peptide backbone, which in turn generates fewer informative ions to unambiguously identify the peptide sequence. A related issue results from the differential nature of this loss from the most commonly modified amino acid side chains, namely serine, threonine, and tyrosine. In principle, fragmentation can occur through three routes, *via* the intact ion, a neutral loss of 80 Da (HPO_3_), or a neutral loss of 98 Da (H_3_PO_4_ or HPO_3_ and H_2_0) [49]. Typically, a loss of 98 Da (H_3_PO_4_) is observed from serine and threonine residues, while phosphotyrosine normally remains intact [50] but can suffer a neutral loss of 80 or 98 Da (HPO_3_ and H_2_O) should there be a nearby side chain bearing a hydroxyl group. The latter neutral loss where there is a concurrent loss of water is an especially difficult situation because this loss can be derived from S/T, making it difficult to distinguish whether the phosphate is present on Y or S/T should there be insufficient product ions available.

Though these are not hard and fast rules, they are often implemented in search engines such as Mascot [34]. Additionally, MS^3^ experiments are performed when neutral losses from phosphopeptides are observed in MS/MS spectra, creating additional ion series from which inference can be made [51]. Finally, it should not be forgotten that frequently the phosphorylated isoform of a given protein might only be present in relatively low amounts, as only low stoichiometries may be necessary for downstream signaling effects. Thus, the quality of signal may be close to or below the sensitivity of the instrument, further hampering the ability of the search engines to detect signal from noise. This was elegantly illustrated by Olsen and colleagues who showed that most phosphosites exhibit less than 10% occupancy during S-phase of the mammalian cell cycle [15].

Knowledge of the relative level of phosphorylation and how it varies in biological systems is therefore clearly valuable information, and quantitative methods are available for phosphoproteomics. Interested readers are referred to an excellent recent review, which covers many of the identification issues also dealt with here [52].

Initially, the only common MS method available for fragmentation of peptides was CID, where precursor ions are subjected to physical collisions, thereby providing the necessary potential energy for fragmentation to occur. Because, as noted, the phosphoester covalent bond is more labile than that of the amide bond, there is a higher chance that the phosphate group is fragmented, hindering the necessary formation of sequence-informative ions. While the characteristic dominant phosphate neutral loss ion is useful in identifying that the precursor is indeed a phosphopeptide, it does not yield enough ions to identify the underlying sequence. Fortunately, alternative activation methods have been made available, which help overcome some of these issues [53].

## 4 Activation methods that improve phosphorylation analyses

Two relatively recent advances that are of utility to phosphoproteomics include the collision-based, multistage activation approach [54,55], where the intact ion following loss of the phosphorylation group is purposely reselected for fragmentation, and high-energy collisional dissociation (HCD) where higher energy is applied than conventional CID [56,57]. While both have been shown to have positive benefits for phosphopeptide identification [58], CID remains a staple activation method in phosphoproteomics because of its superior acquisition speeds that enable more comprehensive coverage of a sample. Hence, with CID more spectra may be acquired, but this does not necessarily lead to the highest number of uniquely identified species, a trade-off between quantity and quality of MS/MS [55,59].

However, a real landmark in the field was the introduction of electron-transfer dissociation (ETD) [60]. This method involves the transfer of an electron to precursor (cat)ions via a radical anion, which invokes the dissociation of amide bonds [60–62].

The advantage over collision-based methods that ETD (and its later derivatives) provides is the capability to bypass labile-biased dissociations associated with some PTMs such as phosphorylation, allowing the modification to remain intact and available for localization calculations. There was, however, a potential downside from an informatics perspective to the use of ETD; search engines were not optimized to process this type of data. All algorithms were originally built and developed with collision-based fragmentation methods in mind and were made ETD compatible by adapting the algorithms to look for c- and z-type fragment ions produced by ETD. Unfortunately, the idiosyncrasies of ETD-derived data such as dominant unreacted and/or charge-reduced precursor ion peaks, which can affect ion selection for the search engine and ETD-exclusive neutral losses were unknown and therefore nullified the identification and subsequent localization performance benefits of ETD [63,64]. Fortunately, many ETD-related behaviors have now been better characterized allowing notable improvements to be made in this area [63–66]. As a result of the combined efforts of the MS community, ETD-based methods are an excellent complementary approach to their collisional counterparts in phosphoproteomic experiments [67–69].

The effectiveness of these activation methods with respect to site localization was recently studied by Savitski and colleagues [58] who assessed the performance of a search engine difference score (Mascot Delta) on the identification of a set of synthetic phosphopeptides under collision-based (CID, multistage activation, and HCD) and ET-based (ETD and electron-transfer dissociation with suppplemental activation [70]) activation methods. Here, they showed superior identification and localization performance for data derived from all the advanced activation methods, most notably HCD and ETD, compared to conventional CID, conforming with the rationale behind using the new activation methods above. This was followed up with a more comprehensive study on a larger peptide library, again confirming the potential for HCD to identify more phosphopeptides and providing an excellent resource for further algorithm development [71].

One can further attempt to enhance peptide identification performance by applying complementary activation strategies where the same precursor *m*/*z* is subject to alternate sequential activation methods. These normally consist of CID/ETD/HCD where any combination (or all) can be used [72] and MS/MS can then be searched individually or merged together for identification. However, the latter choice does apparently present some problems where certain tools, namely database search engines, are not well optimized to deal with activation-composite MS/MS [73].

## 5 More advanced informatic approaches to improve identification

The arsenal of informatic tools available for analyzing data from phosphoproteomic studies could be considered simultaneously as a curse and blessing. With so many tools and their own unique algorithms, one can acquire substantially different results from the same mass spectral data. This was shown in previous studies to affect protein identification in proteomic experiments [74], but the outcome of the ABRF study suggests it might be more severe in the phosphoproteomic realm. However, the variety of different underlying algorithms applied by each search engine offers a parsimonious way to take on this challenge where interrogating the same data from different, orthogonal perspectives provides a simple but robust solution. As noted, this concept has been demonstrated many times in traditional proteomics studies [74–76], and has also recently been shown to reduce of false-positive identifications in phosphoproteomics by combining the output from multiple informatic tools [77].

## 6 Site localization

**Figure 1:**
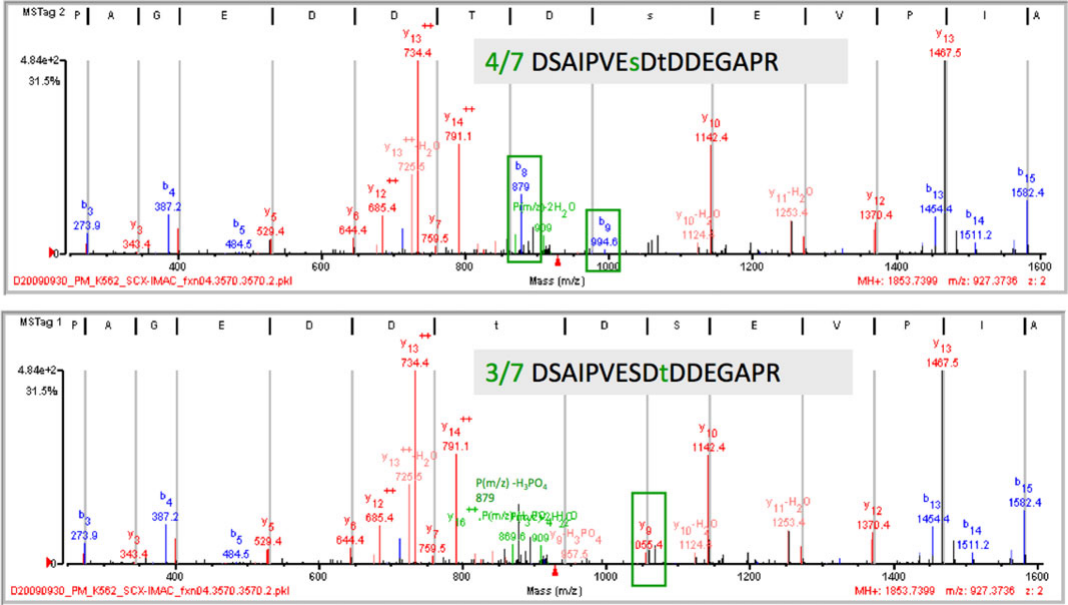
Ambiguity in site assignment of phosphopeptides. The phosphopeptide above generates a product ion spectrum from which it is challenging to unambiguously determine the true site determining ions. In this particular case, two b ions highlighted in green boxes are consistent with serine at position 7 in the peptide being modified, or alternately, the threonine at position 9 could be modified yielding a characteristic y9 ion (green box, lower panel). Experts inspecting the spectrum were divided on which is the most likely interpretation. The possibility that both peptides were present is also not excluded, since they would have the same precursor ion *m*/*z* value (figure adapted from ABRF web site, http://www.abrf.org/index.cfm/group.show/ProteomicsInformaticsResearchGroup.53.htm).

Site localization appears to be a far more challenging task compared to identification, as demonstrated by the iPRG ABRF study, because unambiguous site localization relies on the presence of intact product ions in the product ion spectrum that are characteristic of a given candidate site. To make the problem even worse, site localization becomes considerably more difficult when candidate sites are found in close proximity in the peptide sequence, generating fewer discriminatory ions. As a final testament to difficulty of the problem, the ABRF study highlighted a case where even experienced manual curators could not agree with each other when given the same MS/MS spectrum and the known sequence, shown in Fig.1.

## 7 Site localization algorithms

There are two classes of localization algorithms available to the public: probability-based localizers (PBLs) and search engine difference (SED) scores [78].

## 8 Probability-based localizers

The origin of many PBL tools stems from algorithms originally designed to process MS^3^ mass spectra [79], which were subsequently then applied to the PTM problem [8]. The algorithm designed by Olsen and Mann formulates the localization problem as a binomial probability calculation, attempting to calculate a probability for each candidate phosphosite. In this equation, *k* is the total number of intact phosphorylated ions successfully matched to theoretical ones for a candidate site and *n* is the total number of ions possible. The choice of using intact phosphorylation ions (and not others, such as those derived from neutral loss) during peak annotation is twofold. First, these ions are essential for determining the precise site position and are therefore the most informative. Second, inclusion of other ions may degrade the localization problem by influencing the optimal peak-depth selection and subsequent scoring [80].

The “PTM score” is then computed as the −10 × log_10_ transformation of *P*(*x*):


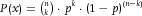
(1)

The probability of matching a peak defines the value of *p* in this model, and equates to 0.04 in this particular instance. This is derived from the “peak depth,” the number of the top most intense fragment ions considered in each 100 *m*/*z* unit bin across the spectrum. In the PTM score algorithm, a peak depth of 4 is used, presuming a 4 in 100 chance of a random match across the 100 *m*/*z* range. This method was employed in the large-scale phosphoproteome study characterizing mammalian signaling networks [8], but was not originally made easily available.

The Ascore algorithm developed a similar probabilistic approach and has become arguably the most well-known gold-standard site localizer in the field [80]. Briefly, the Ascore is composed of two core phases. The first follows Olsen's model, except that a cumulative binomial probability is calculated and the peak depth is selected automatically, attempting to maximize the discrimination between true and false sites. Here, peak depths from one to ten peaks per 100 *m*/*z* unit bins are systematically tested to find which provides the largest increase in discrimination between the first- and second-ranked isoform. The logarithmic transformation of the binomial calculations are termed “Peptide scores” instead of “PTM score” used by Olsen [8]. The second phase applies the same basic workflow to the first, but with one critical adaptation that makes it more suitable for the localization problem, by using solely the “site-determining ions.” These are ions that are exclusive to the phosphopeptide isoform in question and therefore contain one of the candidate modified amino acids. The Ascore is then computed as the difference in peptide score between the first- and second-ranked site candidates.

Since then there have been several localization algorithms based or building on this general principle, summarized in Table1. This includes SLoMo [81], whose most important contribution to this field was that it was the first ET-compatible localizer, highlighting the utility of alternate activation methods for phosphopeptide determination.

**Table 1:** Site localization algorithms

Name	Class of algorithm	Core algorithm	Activation methods supported	Notes and availability	Interface	Prerequisites and/or dependencies	Report alternate sites^a^	References
Ascore	PBL	Cumulative binomial probability	CID	First to implement site-determining ions http://ascore.med.harvard.edu/ (Free)>	Commandline	Requires a pepXML containing PSM information and corresponding MS/MS in individual.dta formatted files	No	[80]
SloMo/TuboSloMo	PBL	Cumulative Poisson distribution	CID, ETD	Was the first PBL available for ETD-derived data http://massspec.bham.ac.uk/slomo/ (Free)	Commandline	Requires a pepXML containing PSM information and corresponding MS/MS in individual.dta formatted files	Yes (Top-2)	[81]
PhosphoRS	PBL	Cumulative binomial probability	CID, ETD, HCD	Has been built and tested on CID-, ETD-, and HCD-MS/MS Version 1.0 http://cores.imp.ac.at/protein-chemistry/downliscoverer; Version 3.1 can be downloaded for ProteomeDiscoverer software	Commandline (v1.0) Vendor (v3.1)	Version 1.0 requires a custom XML format containing both PSM information and corresponding MS/MS. No specific search engine is preferred, as long as the necessary PSM details can be extracted	Yes (All)	[82]
LuciPhor	PBL	Log odds	CID, HCD	First algorithm to implement FLR estimate http://luciphor.sourceforge.net/ (Free)	Commandline	Uses Transproteomic-pipeline (TPP) supported search engines (Mascot, X!Tandem, and SEQUEST/COMET), processed via xinteract to pepXML file. Available under Linux OS	Yes (Top-2)	[86]
MaxQuant PTM score	PBL	Exact binomial probability	All	Also includes site occupancy when quantification information is available, scored based on equation used in Olsen et al. [8] paper http://www.maxquant.org/downloads.htm	GUI	None	Yes (All)	[8]
Mascot Delta	SED	Score difference between first- and second-ranked isomers	All	http://www.matrixscience.com/server.html	Commandline	Mascot.dat files are required. Many groups have written code (including Mascot) to process.dat files	Yes (All)	[58]
ProteinProspector (SLIP)	SED	Score difference between first- and second-ranked isomers	All	http://prospector.ucsf.edu/prospector/mshome.htm	Webserver	Requires (free) registration on ProteinProspector webserver	Yes (all)	[83]
PhosphoScore	Node cost delta between best and second-best candidates	Directed acyclic graph using intensity and mass deviations to weight nodes	CID	https://github.com/evansenter/ucsb/tree/master/school/CS167/main_project/code/PhosphoScore	GUI or commandline	Specific to SEQUEST search engine and explicitly requires.OUT (SEQUEST results) and.dta (peaklists)	No	[104]
PhosphoScan	PBL	Cumulative binomial probability	CID	Available upon request to authors	GUI	Standalone (GUI) tool	Yes	[105]
D-Score	SED	Difference between top- and second-ranked site isomers (posterior error probability (PEP))	All	Standardized localization metric enabling comparison with other search engines. Proof-of-principle paper suggesting the applicability of universal deltas with PEP. No “tool” is currently available	NA	Computation of posterior error probabilities for first- and second-ranked hits required for delta PEP	Yes (all)	[84]
PhosSA	DP (delta between first and second best site candidates)	Dynamic programming using sum intensity of matched site-determining ions to find best site candidates	CID, HCD	http://helixweb.nih.gov/ESBL/PhosSA/	GUI	Compatible with SEQUEST, Mascot search engines and ProteomeDiscoverer	Yes (status assigned to all candidate peptides)	[106]

GUIs: graphical user interfaces.

a) At least second candidate sites are also provided. In principle, all candidate sites are reported by most PBL tools, usually up to and including a maximum of two sites per peptide.

Another algorithm of note is PhosphoRS [82], which further develops the concept of peak-depth determination. This is an important aspect of site localization since the quality of the peak annotation and subsequent selection of the most suitable and informative ones underpin subsequent scoring algorithms. This is an issue with all site localizers (and other computational proteomic tools) that apply intensity-based filters to reduce chemical or instrumental noise but may inadvertently eliminate truly informative peaks [78]. In the context of PBLs, all algorithms prior to PhosphoRS apply this partitioned *m*/*z* unit strategy to annotate intact ions with a predefined [8] or optimized but static peak depth [80,81]. Because some areas in a theoretical MS/MS spectrum may be denser, one should use a larger peak depth in denser regions and vice versa in sparser ones. PhosphoRS addresses this issue by allowing the peak depth to vary according to the local peak density in regions of a MS/MS spectrum, resulting in different estimates for probability *p* for matching a single fragment ion in Eq.(1) for each spectrum considered. Equally, it will vary according to instrument and fragmentation methods, supporting both low- and high-resolution instruments. The search engine integrated in to the MaxQuant suite, Andromeda [32], also uses a similar approach to dynamically select peaks, thereby improving spectrum annotation, and applies this in their own version of the PTM score. The authors suggest this leads to superior performance in detecting multiply modified peptides.

## 9 SED scores

All search engines consider candidate PSMs in rank-ordered lists to assign confidence and help determine the most likely match. A key principle embodied in the first automatic spectrum search tool, SEQUEST, has been exploited for phosphoproteomic localization purposes too, namely that the top hit should score significantly higher than the second-ranked hit if it is truly correct. The higher the quality, the greater the score difference and more confident the identification (or in this case, localization). SED scores are computed in the situation where multiple sites are possible for a given modification and the first- and second-ranked candidates are PTM isomers of each other.

These approaches have proven popular due to their simplicity and can be applied, in principle, to any scoring method. Indeed, most of the tools available to compute this type of score are linked to particular search engines. Examples of SEDs for localization include Mascot Delta [58] and site localization in peptides (SLIP) [83], developed for the Mascot and ProteinProspector search engines respectively, both of which have been shown to offer good performance in distinguishing alternative phosphorylation sites based on the search engine scores [58,66,83]. Recently, a search engine independent delta score named the “D-score” was published, which converts search engine delta scores into posterior error probabilities [84]. This approach has some practical advantages over a single SED; first, standardizing a search engine specific localization delta score such as Mascot Delta and SLIP will place them into a unified scoring framework, thereby allowing direct and valid comparisons. The second is that a standardized localization metric supports more advanced combinatorial methods, similar to those already in use for multiple search engine peptide identification purposes that demonstrate improvements over single stand-alone search engines [74,75,85]. It remains to be seen, however, whether SED-based approaches can outperform the theoretically more rigorous PBL methods.

## 10 The status of the false localization rate

An additional issue facing the proteomics practitioner undertaking phosphoproteomics experiments is when to believe a set of phosphosite assignments when ambiguity exists, that is, in multisite peptides. For identifications, the field has developed FDR-based approaches, but as Chalkley and Klauser pointed out [78], for site assignment we need a false localization rate or FLR. The FLR is the localization equivalent of the FDR for peptide identification; a method to estimate, and therefore control, the proportion of falsely localized sites through a target-decoy strategy. However, it is not immediately obvious how to estimate such a rate. As noted by Chalkley and Klauser [78], not only is the FLR sort after for this reason but also because it would allow a direct and fair comparison of different site localization tools, and provide a universal metric to measure against (and potentially to integrate multiple tools in a principled way).

At present, there is no universally accepted method to determine the FLR. The key hurdle here being how to define the decoy population from which a background, null distribution of scores can be estimated, thereby enabling an FLR to be estimated. PhosphoRS developed a tool-specific estimate of this property, using synthetic peptides of known phosphorylation status to calculate empirical FLRs, and suggesting a PhosphoRS probability of 0.99 equates to an FLR of 1%. In more general terms, it has been proposed to generate decoy instances by theoretically phosphorylating residues that do not carry this modification in nature. Here, Chalkley reasoned that appropriate decoy residues should have a similar frequency and close proximity to real phosphorylatable (STY) residues and suggests the use of proline and glutamic acid, which correlate with serine and threonine, respectively, in the general context of these criteria [78,83].

In 2013, Fermin and colleagues achieved a milestone in the field where they presented the LuciPHOr algorithm [86], the first algorithm to include a formal FLR estimation. Using a synthetic set of phosphopeptides [58], allowing the calculation of the true FLR, they demonstrate similar or superior performance in terms of site assignment compared to Ascore and Mascot Delta. Like other algorithms, LuciPHOr considers all phosphorylatable residues as candidates, but also considers all non-native phosphorylation sites as decoys to estimate an FLR rate. Their scoring procedure compares the relative distributions of fragment ion intensity and mass accuracy for the candidate, annotated phosphopeptide (for each given phosphopermutation) to nonannotated (random) peaks. The greater the separation between the two populations, the better the score, which in this instance is a log-odds score generated from the two. A delta score is then computed between the best and second-best phosphopermutation. For the FLR calculation, the necessary target and decoy distributions are derived from the best target and decoy LuciPHOr delta scores, respectively. It will be interesting to note how this score performs and whether the FLR can be influenced by database size and nature, as has been noted for FDR (e.g. [87,88]). The current version of LuciPHOr is compatible with most of the popular search engines and their scoring metrics, including PeptideProphet (*p*-values), X!Tandem (translated e-values), Mascot (ion scores), and SEQUEST/COMET (Xcorr), and presently works with CID and HCD-derived MS/MS. It has been integrated with the Trans-Proteomic Pipeline [89].

## 11 Isomers and the problems they pose

The localization problem is further impeded by isomeric species where the sequence and phosphorylation status are identical but the location of the site is different, for example, in the following two sequences: ANSLMSpSQFGK and ANSLMpSSQFGK (where pS = phosphorylated serine). Not only are the masses (or *m*/*z* values) of the isomers identical, but they are also likely to have identical physicochemical properties, and fail to separate during the LC. Additionally, it has been shown that phosphates can switch between side groups in the gas phase under certain conditions, generating artifactual isomer pairs [90]. In the former case, the isomers are likely to coelute and in both cases lead to the generation of a chimeric MS/MS spectrum, the extent of which depends on the severity of chromatographic overlap. This is detrimental to localization on two accounts. First, the majority of site localization algorithms are based on the assumption that there is only one correct phosphorylated form and employ a difference-based scoring scheme that relates the deviation between top-ranked candidates to assign confidence in site localization. In the isomer situation where fragment ions belonging to the true alternate sites coexist, confident localization becomes far more difficult as they would naturally diminish the delta. Fortunately, in the case of artifactual isomer pairs, it appears this situation does not detrimentally affect localization analysis because the event is relatively rare resulting in fewer product ions derived from rearranged species. As a result, such ions fail to pass the intensity-based filters of localization algorithms [91].

The second problem is related to the standard instrumental setup of most phosphoproteomic experiments. In order to maximize coverage and minimize redundancy, MS experiments will typically exploit a dynamic exclusion period where previously selected precursor ions are not reselected for fragmentation until a user-defined period of time has elapsed. Depending on the degree of coelution between isomers, it is possible that subsequent isomers are not selected for fragmentation if the exclusion window is long enough.

Fortunately, the occurrence of such species has been estimated to be low, approximately 3–6% of all potential phosphopeptides [92]. However, although this value is low, there are presently no publicly available localization algorithms that integrate elution time information to detect potential coeluting isomers into their scoring scheme, so such many of these species may be lost and pass undetected through the mass spectrometer. To counter this, Courcelles and colleagues have developed algorithms that help distinguish between separated, partially coeluted, and overlapping phosphorylation species, with some success [92]. They do suggest, however, that ultimately targeted MS strategies will be necessary to detect these additional isomeric species after preliminary data-directed acquisition studies. It is also perhaps worth noting that such isomeric species might also be functionally indistinguishable in biological terms, reducing the potential severity of this issue, if the effect is generated from modification of either of two adjacent sites.

## 12 Computational phosphoproteomics in practice

As noted here, there is a wide choice of algorithms on offer for the problem of site localization. While each has been of value to the field, one important aspect has not yet been discussed; is it straightforward to acquire and implement the algorithm? This is an important question to address for the user community, where unless it has been adopted by a vendor and incorporated directly into commercial software, the algorithms may not be suitable for noninformaticians. In particular, some tools may need to run *via* the command line and have very specific prerequisites or dependencies, including vendor-specific libraries, before they can be used. Typically, these include sequence identifications from a database search engine and the MS/MS corresponding to said identifications (depending on the type of localizer), all of which need to be provided in a specific format. Failure to meet any of these criteria might prevent the tool from generating the desired output, necessitating informatic support to provide a fix. However, even with dedicated informatics support the process of setting up and running software can be challenging, for example, when inadequate instructions for installation are supplied or where the software requires files whose formats are now obsolete. Clearly, the use of standardized and consistent, community-supported file formats [93] makes this problem far simpler. A good example is the Transproteomic pipeline (TPP) workflow, which contains executables for converting Mascot, X!Tandem, COMET, OMSSA, and SEQUEST output into.pep.xml. One could envisage writing a single parser that universally deals with.pep.xml to provide return delta scores from all these search engines. Similarly, we recommend interested users explore some of the following workflows packaged with user-friendly graphical user interfaces for noninformaticians for handling PTM and localization scores; notable examples include PeptideShaker [94], MaxQuant [95], and PTMProphet [89]. Alternatively, one can use web servers such as ProteinProspector whose service provides the SLIP score where users can easily acquire identification and localization scores in a tabulated format.

## 13 Data standards supporting phosphoproteomics

As noted above, file formats can be a barrier to integration of proteomic data types and sharing with colleagues. This is true for phosphoproteomic data too, and community-driven standards present a useful way to surmount this barrier. The Proteomics Standards Initiative (PSI) has been developing standard data formats, as well as minimum reporting guidelines for proteomics for many years. Relevant standards include mzML for raw MS data or peak lists [96], mzIdentML for peptide and protein identification data [97] (e.g., the output of search engines), and mzQuantML for quantitative data [98], used as an internal, input, or output format to quantitative software. In this context, most search engines support a search in mzML as input—which functions equally for traditional as well as phosphoproteomics studies. In terms of the output of search engines, several search engines natively support an export of mzIdentML, and for many other search engines, file format converters exist. The growing set of implementations for mzIdentML (and mzML) is important, since, as an example, ProteomeXchange consortium databases support these PSI standards as an input and a format for downloading results [99]. The standards also facilitate open source development, so that informatics groups can build pipeline approaches, without needing to consider writing many different file format converters.

The stable, supported release of mzIdentML is version 1.1. Due to the design of the standard, scores or probabilities associated with modification site localization are challenging to encode systematically in the format. The PSI working group has been working toward an update to mzIdentML (version 1.2), which is undergoing the final stages of revision, and will be released later in 2014—including updated guidelines for protein inference [100] and solving various other open issues with the standard. The mzIdentML 1.2 update will have minimal changes to the core XML Schema of the standard, but will provide a consistent way of representing site localization scores using controlled vocabulary terms, which can be checked by the validation software [101]. The update should be of significant benefit to the informatics community working on phosphoproteomics tools, since as mentioned above, there are issues with stand-alone tools accepting incompatible file formats. Once released, mzIdentML 1.2 can function as an input and output format for such tools, as well as acting as an output format from search engines that natively perform site localization, for example, for upload into ProteomeXchange. It is likely that the same mechanism for encoding site localization scores will be adopted in mzQuantML, if sufficient need arises to encode such ambiguity alongside quantitative data about phosphopeptides (e.g.).

The three standards described (mzML, mzIdentML, and mzQuantML) are all developed in XML (Extensible Markup Language) and capture relative complex data about different stages of a proteomics pipeline. While tutorials exist describing how lab scientists and developers can use the standards [102], it is acknowledged by the PSI that the XML-based standards can be challenging to work with for nonexpert groups. As such, the PSI has recently developed a text-based, tab-separated standard called mzTab that is considerably simpler than the other standards [103]. mzTab is designed for loading directly into spreadsheet or statistical software, capturing a summary of identification and quantification results, potentially in the same file. mzTab has native support for capturing site localization scores associated with a given peptide identification, and tools are starting to emerge that export into mzTab format. It is likely that mzTab will be accepted as an input to ProteomeXchange (and format for downloading results) from ProteomeXchange in the near future.

## 14 Concluding remarks and future outlook

The informatics analysis of phosphorylation sites in proteins has proven to be a difficult task from both the identification and, even more so, localization perspectives. However, concerted efforts from the field have helped develop a range of integrated experimental and informatics solutions to enable phosphoproteomics to capture snapshots of cellular regulation *via* MS. Indeed, many labs are now able to generate fully quantitative phosphoproteomic datasets [11,15], an aspect we have not covered in this review.

Experimentally, increasingly advanced activation methods have been introduced that have greatly aided the field, each by circumventing weaknesses associated with CID making the informatics substantially more effective. Informatically, while there has been an expansion in the number of localizer tools available, they mostly employ the same logical scoring schemes so limited progress has been made in this area. The advent of a search engine independent score by Vaudel and colleagues [84] advances the possibility of applying a multilocalizer approach, taking advantage of the inherited orthogonality of each SED, and complementing multisearch engine approaches already in evidence in standard high-throughput proteomics.

Perhaps the most prominent remaining hurdle to overcome is the lack of a widely accepted method to control for false-positive localizations. The recent emergence of Fermin's work to compute the FLR maybe the necessary catalyst toward solving this problem, either through the creation of a new generation of site localizers or adoption of an FLR scheme into existing tools.

In conclusion, although the informatics of phosphoproteomics remains challenging, sufficient progress and tools are available to enable motivated scientists to characterize and address their system of interest.

## Abbreviations

ABRF: Association of Biomolecular Resource Facilities
ETD: electron-transfer dissociation
FDR: false discovery rate
HCD: high-energy collisional dissociation
iPRG: Proteome Informatics Research Group
PBLs: probability-based localizers
PSI: Proteomics Standards Initiative
PSMs: peptide spectrum matches
SED: search engine difference
SLIP: site localization in peptides
